# Anemia in Pediatric Kidney Transplant Recipients—Etiologies and Management

**DOI:** 10.3389/fped.2022.929504

**Published:** 2022-06-20

**Authors:** Anne Kouri, Shanthi Balani, Sarah Kizilbash

**Affiliations:** Department of Pediatrics, University of Minnesota, Minneapolis, MN, United States

**Keywords:** pediatric kidney transplant, anemia, erythropoietin, iron deficiency, hepcidin

## Abstract

Posttransplant anemia (PTA) is a common complication of pediatric kidney transplantation, with a prevalence ranging from 22 to 85%. PTA is categorized as early (within 6 months posttransplant) and late (>6 months posttransplant). Early PTA is typically associated with surgical blood losses and iron deficiency. Late PTA primarily results from graft dysfunction; however, iron deficiency, drug toxicity, and posttransplant inflammation also play a role. PTA is more severe compared with the anemia in glomerular-filtration-rate matched patients with native chronic kidney disease. Treatment of PTA is directed toward the underlying cause. Erythropoiesis stimulating agents (ESA) are effective; however, their use is limited in the transplant setting. Timely diagnosis and treatment of PTA are vital to prevent long-term adverse outcomes in pediatric transplant recipients.

## Introduction

Studies in pediatric transplant recipients have demonstrated a varying incidence of PTA ranging between 22 and 85% depending on the post-transplant time period being measured and definition of PTA in various studies (1–7). The incidence of PTA is higher in the immediate transplant period (1). PTA is associated with several adverse outcomes in adult and pediatric populations (4, 8–13). Adult data indicate lower patient and graft survival and a lower quality of life in patients with PTA (12–14). A retrospective study of 3,669 pediatric kidney transplant recipients corroborates adult findings, demonstrating an association between PTA and an increased risk of graft failure (*P* = 0.01) or combined graft failure and death (*P* < 0.01) (4). Furthermore, pediatric studies among children with chronic kidney disease indicate an association between anemia and left ventricular hypertrophy (10, 11). Despite its high prevalence and deleterious long-term consequences, clear pediatric guidelines for evaluation and management of PTA are lacking, and the optimal hemoglobin after kidney transplantation remains unknown.

PTA is divided into two categories: early PTA (within the first 6 months) and late PTA (>6 months posttransplant) (15). Early PTA is primarily due to postsurgical blood losses and iron deficiency. Late PTA is largely caused by reduced graft function; however, drug toxicity, iron deficiency, posttransplant infections, and inflammation also contribute. Other contributing factors include erythropoietin resistance, perturbations in the FGF23 pathway, and vitamin D deficiency. Herein we review the mechanisms and management of PTA in children.

## Acute Blood Loss and Iron Deficiency

Iron deficiency is common in the posttransplant population and plays a significant role in PTA. Numerous factors, both pre- and posttransplant, predispose patients to the development of iron deficiency (16). Advanced chronic kidney disease (CKD) in the pretransplant period is associated with low iron reserves due to nutritional deficiencies, increased iron utilization from erythropoiesis-stimulating agent (ESA) use, and chronic blood loss during hemodialysis (17). The immediate posttransplant period is characterized by an early peak in erythropoietin production, which is triggered by low tissue oxygen and graft ischemia (18). This increases erythropoiesis and depletes iron stores further. To compound this, the post-operative state of oxidative stress and inflammation increases production of IL-6 and hepcidin, which in turn inhibit intestinal iron absorption and release from the reticuloendothelial system, resulting in a functional iron deficient state (18–20).

Perioperative blood losses, including surgical blood loss and frequent phlebotomies in the posttransplant period, exacerbate the state of iron deficiency. While uncomplicated transplant surgeries do not typically result in significant blood loss, the magnitude and duration of pretransplant azotemia may aggravate the blood loss during surgery (21). A study conducted among adult transplant recipients estimated an average blood loss of 780 ml, roughly 295 mg of iron, through phlebotomies during the first 12 weeks, assuming 5–7 ml per vial blood draw (22). Most pediatric centers have more frequent monitoring than the adult study's weekly monitoring, increasing the risk of iron losses in the peri-transplant period.

It does seem that iron deficiency is almost certain to develop in the posttransplant period. In fact, an adult study discovered that 60% of transplant recipients who did not have an iron deficiency at the time of transplant developed it by 6 months (23). Likewise, iron deficiency at the time of transplant remained persistent even 6 months after transplant without intervention (24). Adult studies report the prevalence of posttransplant iron deficiency to be 20–50% (22). Congruent with the adult data, a single-center cross-sectional pediatric study from Australia found iron deficiency as the cause of PTA in 34% of recipients (2). Consensus is lacking regarding the optimal definition of iron deficiency in pediatric kidney transplant recipients. The prevalence of iron deficiency reported in studies varies by definition used. For instance, a single-center pediatric study by Yorgin et al. with 162 patients over 10 years found that most patients (80–100%) had evidence of iron deficiency when the CKD definition for iron deficiency was used (serum iron <50 mcg/dl and ferritin <100 mg/ml), however, only 27–56% were categorized as iron-depleted when normative values for healthy children were used (1). Additional studies are required to determine the optimal definition of iron deficiency in pediatric transplant recipients.

Although iron deficiency is typically associated with early PTA, it also plays a significant role in late PTA. Yorgin et al. found that high rates of iron deficiency persisted up to 60 months post-transplant (1). This is corroborated by an adult cohort study from Israel where iron deficiency contributed to 35% of late PTA, (25), and another study from the Netherlands where the prevalence of iron deficiency anemia was 13%, and iron deficiency without anemia was 30% (26). According to adult data, iron deficiency is associated with an increased risk of mortality, independent of anemia and GFR (26). Despite this, iron deficiency is not routinely monitored, and iron supplementation and erythropoietin are typically discontinued after the transplant.

The management of posttransplant iron deficiency is complicated by uncertainty about the target hemoglobin, iron deficiency measures such as ferritin or transferrin saturation, and the role of erythropoietin. In the absence of specific recommendations from KDIGO or KDOQI, the consensus remains to use iron deficiency criteria in the CKD population of serum ferritin of <100 ng/mL and TSAT of <20 (9). There is a growing interest in the study of novel makers such as reticulocyte hemoglobin content, percentage of hypochromic red cells, and soluble transferrin receptor to diagnose iron deficiency, however, additional research is required to determine their utility in the pediatric transplant population (27).

Iron deficiency can be treated with parenteral or enteral iron; enteral iron is typically preferred in this outpatient population. If enteral therapy is unsuccessful, it is reasonable to switch to parenteral iron, which is well tolerated with no increase in adverse events, as demonstrated in adult studies (22, 28). Parenteral iron may also be more effective at replenishing iron stores, given the hepcidin-mediated poor intestinal absorption in the setting of posttransplant inflammation.

## Anemia of Chronic Kidney Disease

Anemia is a well-recognized complication of CKD. Although it results from a complex interplay of factors, erythropoietin deficiency due to decreased synthesis by renal peritubular capillary interstitial cells and iron deficiency play a predominant role (29, 30). Additional mechanisms include B12 and folate deficiencies (8) functional iron deficiency due to increased hepcidin caused by reduced urinary excretion and uremic inflammation (31, 32), reduced lifespan of red blood cells due to uremia-associated oxidative stress (33), and hyperparathyroidism associated bone marrow fibrosis (34).

Although advances in immunosuppression and transplant care have significantly improved patient and graft survival, graft dysfunction is inevitable. A cross-sectional study of 45 pediatric kidney transplant recipients found a 62% prevalence of CKD stage 3 or higher (5). Although graft dysfunction is a significant cause of PTA, adult and pediatric data indicate a higher prevalence and greater severity of PTA compared with native CKD for the same level of kidney dysfunction (5, 35, 36). In a study of 147 pediatric patients, White et al. found higher odds of developing anemia at all stages of CKD in transplant recipients vs. patients with native CKD (5). Similarly a recent pediatric study by Oruc et al. showed lower hemoglobin (*p* = 0.04) despite a higher erythropoietin level (*p* < 0.001) in transplant recipients compared with GFR-matched native CKD patients (35). These data suggest that additional mechanisms are at play in the posttransplant population.

Adult and pediatric studies demonstrate the efficacy of ESAs in correcting PTA without increasing adverse side effects (14, 37–39). In addition to treating erythropoietin deficiency caused by graft dysfunction, ESAs combat erythropoietin resistance seen in the transplant setting. The posttransplant erythropoietin resistance is commonly attributed to chronic inflammation. Other contributors include functional or absolute iron deficiency and ongoing hyperparathyroidism. Erythropoietin resistance is known to occur even with normal graft function, placing patients at risk of PTA (40). Despite the benefits of ESA therapy, it is underutilized in pediatric transplant recipients. A pediatric cohort from ESPN/ERA-EDTA registry found that only 10% of patients were prescribed ESA despite a PTA prevalence of 50% (4).

KDIGO and KDOQI recommend using the Guidelines on Anemia in CKD to evaluate and manage PTA. Authors would argue that these guidelines are inadequate for PTA given its multifactorial nature and greater severity. An open-label, multicenter, randomized controlled trial in adult kidney transplant recipients showed that complete correction of anemia (hemoglobin >13g/dL) using ESAs improved graft survival and quality of life without increasing the adverse effects (38). In addition to indicating the efficacy of ESA in PTA, this study argues that the optimal target hemoglobin in transplant recipients may be higher than that in the GFR-matched CKD population. The KDIGO and KDOQI guidelines for CKD anemia may not be fully applicable to transplant recipients. Additional transplant-specific studies, including those examining outcomes in pediatric populations, are needed to develop transplant-specific guidelines.

## Anemia of Chronic Disease

Anemia of chronic disease, also known as anemia of inflammation, occurs in patients with acute or chronic inflammatory conditions such as infections, malignancies, autoimmune diseases, and chronic kidney disease (41). It is characterized by normocytic anemia that results from several mechanisms, including dysregulated iron homeostasis, blunted erythropoietin production, erythropoietin resistance, and reduced red blood cell life span (42, 43).

The inflammatory cytokines increase hepcidin, an iron regulatory protein. Hepcidin is a 25-amino acid antimicrobial peptide produced by hepatocytes and, to a lesser extent, adipose tissue and macrophages (44, 45). It causes degradation of the sole cellular iron exporter, ferroportin, present on the surfaces of enterocytes, macrophages, and hepatocytes, thereby reducing intestinal iron absorption and bodily iron mobilization—the latter results in a functional iron deficiency and limits iron availability for erythropoiesis (31). Hepcidin is stimulated by iron overload and inflammation (specifically interleukin-6) and inhibited by iron deficiency, hypoxia, and erythropoietin-stimulated erythroblasts (8). Therefore, hepcidin levels increase in states of iron overload and inflammation and decrease in states of iron deficiency, hypoxia, and times of increased red cell production (45, 46). Hepcidin levels also increase with kidney dysfunction/CKD due to reduced urinary excretion (8). In kidney transplant recipients, hepcidin levels are affected by many factors, including inflammation, kidney dysfunction, and iron overload.

Kidney transplantation represents a physiologic state of inflammation (47–49), as indicated by a retrospective study of 128 pediatric kidney transplants that demonstrated elevated high-sensitivity C-reactive protein and ESR levels in 30% of the recipients (7). Potential etiologies of posttransplant inflammation include immune activity against the allograft, acute and chronic infections, and graft dysfunction resulting in reduced urinary excretion of pro-inflammatory cytokines and accumulation of uremic mediators of inflammation (50, 51). In addition to causing the hepcidin-mediated functional iron deficiency, posttransplant inflammation causes resistance to erythropoietin.

The treatment of anemia of chronic disease in transplant recipients is directed toward the underlying cause, such as infections and rejection. Iron supplementation to overcome hepcidin-mediated functional iron deficiency and ESA administration may be beneficial (42). Targeted therapies against hepcidin are currently limited (42). Roxadustat, an hypoxia-inducible factor (HIF) prolyl hydroxylase inhibitor, which mimics the body's response to hypoxia, has been shown to lower hepcidin levels in CKD-related anemia (52). This medication is not approved by the Food and Drug Administration (FDA) in the United States; however, it is approved for use in adults in China, Chile, Japan, South Korea, and Europe.

## Drug-Induced Anemia

As described above, transplant recipients exhibit a higher prevalence and greater severity of anemia compared with GFR-matched non-transplant CKD patients. As listed in Table 1, posttransplant medications significantly contribute to this difference in severity.

**Table 1:** Drug-induced anemia in kidney transplant recipients.

**Posttransplant medications**	**Comments**
Thymoglobulin	PTA *via* bone marrow suppression (53) May cause hemolytic anemia (54)
Mycophenolate Mofetil	Causes generalized bone marrow suppression Pure red cell aplasia may be seen (55) Anemia may be associated with genetic polymorphism (56)
Azathioprine	PTA *via* bone marrow suppression Macrocytosis may occur Pure red cell aplasia may be seen (57, 58) Thiopurine methyltransferase (TPMT) and nudix hydrolase 15 (NUDT15) gene mutations are risk factors for profound pancytopenia (59)
Tacrolimus	Pure red cell aplasia may be seen (60) May cause acute hemolytic anemia (61) Associated with thrombotic microangiopathy (41, 62)
Cyclosporine	Associated with thrombotic microangiopathy (62, 63)
mTOR inhibitors	Causes anemia in a dose-dependent manner (63, 64) May induce profound red blood cell microcytosis (65) May cause thrombotic microangiopathy (66)
Belatacept	Anemia is a common side effects (67)
Rituximab	Anemia seen in 3% of patients (66)
Ganciclovir/valganciclovir	PTA *via* bone marrow suppression Myelotoxicity is greater with valganciclovir
Valacyclovir	Anemia reported in 11–14%
Trimethoprim-sulfamethoxazole	Associated with pure red cell aplasia (68)
Dapsone	PTA *via* bone marrow suppression Associated with dose-related hemolytic anemia (69)
ACE/ARB	Induce anemia in a dose-dependent manner (63)

Posttransplant medications contribute to both early and late PTA. A retrospective study of 3,669 pediatric kidney transplant recipients, with data in ESPN/ERA-EDTA Registry, found the lowest hemoglobin level in the first 6 months posttransplant (4). This is likely because immunosuppressive doses are the highest, and patients are more likely to be on valganciclovir for CMV and trimethoprim-sulfamethoxazole for *Pneumocystis carinii* prophylaxis during the first 6 months.

The antiproliferative medications, mycophenolate and azathioprine, are most commonly implicated in PTA. Both agents are associated with a generalized bone marrow suppression; however, pure red cell aplasia may also be seen (55, 57). The degree of anemia may be more severe with mycophenolate compared with azathioprine (4). The mammalian target of rapamycin (mTOR) inhibitors also cause anemia, characterized by profound microcytosis, *via* bone marrow suppression in a dose-dependent fashion (63–65). Although calcineurin inhibitors do not typically cause bone marrow suppression, pure red cell aplasia has been reported with tacrolimus (60). Furthermore, both tacrolimus and cyclosporine are associated with thrombotic microangiopathy (62, 63). Thymoglobulin also suppresses the bone marrow leading to early PTA (53). Additionally, thymoglobulin is associated with hemolytic anemia due to cross-reacting antibodies (54).

Other medications that cause PTA include renin-angiotensin-aldosterone inhibitors (ACE/ARB). The ACE/ARBs induce anemia by inhibiting erythropoiesis in a dose-dependent manner (63). Prophylactic agents including ganciclovir, valganciclovir, trimethoprim-sulfamethoxazole, and dapsone also cause anemia by suppressing the bone marrow (70).

The management of drug-induced anemia entails decreasing the dose or discontinuation of the offending agent, if possible. ESAs may be used to treat drug-induced anemia (71, 72).

## Other

### Infection-Related Anemia

Although infections may indirectly cause anemia by creating an inflammatory environment, several posttransplant infections directly cause PTA by suppressing the bone marrow. Most commonly, these infections include CMV, EBV, and parvovirus B19 (9). Bone marrow suppression has also been reported with BK infection in rare instances (73). Other infections that may suppress the bone marrow include HIV, hepatitis B, hepatitis C, and HSV (70).

### FGF-23

Fibroblast growth factor-23 (FGF-23), a hormone primarily produced in the osteocytes and osteoblasts in long bones, promotes phosphorus excretion in the urine (74–76). Large adult studies have shown that increased FGF-23 levels are associated with anemia in patients with CKD (77, 78). In the aforementioned retrospective study by Limm-Chan et al. of 59 pediatric kidney transplant recipients, multivariable linear regression analysis found an inverse correlation between total FGF-23 levels and hemoglobin standard deviation score (SDS) (79). However, in the univariate analysis, intact FGF level was not associated with hemoglobin SDS.

### Vitamin D Deficiency

Studies show an association between vitamin D deficiency, low hemoglobin, and ESA resistance. The association between vitamin D and anemia is independent of PTH and is likely due to the immune-modulatory effects of vitamin D (80). Immune cells express vitamin D receptors, which, when activated, inhibit the expression of inflammatory cytokines (80). Correspondingly, Vitamin D deficiency stimulates the immune cells to produce cytokines, resulting in higher hepcidin levels and consequent functional iron deficiency and erythropoietin resistance (81, 82).

## Discussion

Anemia is a well-recognized complication of CKD. Although erythropoietin levels improve after a successful kidney transplant, anemia persists in a large number of pediatric recipients. PTA deserves careful attention as it is associated with several adverse outcomes, including fibrosis on protocol biopsies (7), graft dysfunction, mortality, poor growth, left ventricular hypertrophy, neurocognitive deficits, and a lower quality of life. Despite its high prevalence and adverse outcomes, PTA is frequently overlooked. An adult study of 240 transplant recipients found that among patients with hematocrit <30%, only 36% completed iron studies, 46% received iron supplementation, and 40% received ESA (83).

Early PTA is intricately linked to a patient's pretransplant health. It is vital to ensure that patients have adequate iron stores prior to transplant. Parenteral administration should be considered if they are resistant to enteral supplementation, even in non-hemodialysis settings. Similarly, other nutritional deficiencies that contribute to anemia, such as folate, B12, carnitine, and copper, should be treated before transplant. A recent prospective German study including 146 pediatric patients demonstrated that pediatric kidney transplant recipients treated with recombinant growth hormone prior to transplant had less anemia than those who were not (*p* = 0.05) (84). Hence, pretransplant growth hormone therapy should be offered to eligible patients. Since vitamin D deficiency is a risk factor for anemia, vitamin D levels should be optimized prior to transplant. Considering the high risk of iron deficiency in the early posttransplant period, prescribing iron supplementation to most recipients in the immediate posttransplant period, regardless of the iron stores at the time of transplant, may be reasonable. Iron surveillance should be protocolized for transplant recipients as it is for CKD patients to ensure a timely diagnosis. However, caution should be exercised in patients with high ferritin (>500) and low iron stores as may be seen in settings of inflammation due to concerns about oxidative damage and risk of infections with parenteral iron (85, 86).

Late PTA is usually related to graft dysfunction; however, other risk factors such as iron deficiency, medication toxicity, and infections should be investigated and treated. Antiproliferative agents are the most likely drug-related cause of late PTA. KDIGO recommends monitoring the area under the curve (AUC) for mycophenolic acid and dose reduction of mycophenolate or azathioprine as initial steps (87). However, immunosuppression reduction needs to be balanced against the risk of rejection.

Existing evidence favors ESA use for PTA, regardless of whether it is related to graft dysfunction, erythropoietin resistance, or drug toxicity. In addition to correcting anemia, ESA may reduce fibrogenesis by inhibiting epithelial to mesenchymal transition (38). Considering the posttransplant persistence of pretransplant anemia and posttransplant erythropoietin resistance, it may be reasonable to continue ESA after transplant until early PTA recovery. A retrospective study of 482 pediatric kidney transplant recipients documented a blood transfusion in 39% of recipients within the first month and 48% after the first 12 months posttransplant. The study also found a temporal increase in the rates of posttransplant blood transfusions, illustrating the increasing prevalence and severity of PTA (88). Timely institution of ESA therapy may reduce the frequency of blood transfusions, reducing the risk of graft loss and alloimmune sensitization (37). ESAs may pose logistical challenges given their expense, need for insurance approval in addition to the need for frequent injections, which often make them an unattractive option to pediatric patients and their caregivers. ESAs also require frequent hemoglobin monitoring given the risk of polycythemia and may also be associated with worsening hypertension (89). More extensive studies are needed to determine the optimal dosing and safety of ESA for the treatment of pediatric PTA.

To conclude, anemia is prevalent in pediatric kidney transplant recipients. Despite the disease burden and associated adverse outcomes, PTA does not receive the same attention as anemia of CKD. Additional studies and standardized guidelines are warranted to inform the evaluation of PTA risk factors, optimal target hemoglobin, and the role of ESA (timing and dosing) in the treatment of PTA in children.

## Author Contributions

AK: developed the concept, literature search, and manuscript writing. SB: literature search and manuscript writing. SK: literature search, manuscript writing, and overall editing. All authors participated in the literature search and manuscript writing.

## Conflict of Interest

The authors declare that the research was conducted in the absence of any commercial or financial relationships that could be construed as a potential conflict of interest.

## Publisher's Note

All claims expressed in this article are solely those of the authors and do not necessarily represent those of their affiliated organizations, or those of the publisher, the editors and the reviewers. Any product that may be evaluated in this article, or claim that may be made by its manufacturer, is not guaranteed or endorsed by the publisher.
